# Exploring the Differences in Pneumocystis Pneumonia Infection Between HIV and Non-HIV Patients

**DOI:** 10.7759/cureus.27727

**Published:** 2022-08-06

**Authors:** Mohamed Nasr, Amad Mohammad, Mosab Hor, Ahmed M Baradeiya, Hodan Qasim

**Affiliations:** 1 General Internal Medicine, Mansoura General Hospital, Mansoura, EGY; 2 Hematology and Oncology, Saint James School of Medicine (SJSM), New York, USA; 3 Ophthalmology, Children’s Retina Institute, Los Angeles, USA; 4 Ophthalmology, Palestinian Medical Council, Ramallah, PSE; 5 Research, Fresno Clinical Research Center, Fresno, USA; 6 Internal Medicine, Alfaisal University, Riyadh, SAU

**Keywords:** outcome, prophylaxis, treatment, epidemiology, non-hiv, hiv, pneumocystis jirovecii pneumonia

## Abstract

*Pneumocystis* pneumonia (PCP) is one of the most common opportunistic infections worldwide that affects the lung. *Pneumocystis* leads to pneumonia, caused by *Pneumocystis jirovecii*, formerly known as *Pneumocystis carinii*. In recent decades, PCP has been a major health problem for human immunodeficiency virus (HIV) patients and is responsible for most of mortality and morbidity. However, the increasing number of immunosuppressive-related diseases has led to outbreaks in other patient populations, raising the concern for PCP as it becomes a major concern among those patients. These changes led to marked changes in the prevalence and mortality rates of PCP. Huge variations in those parameters among HIV and non-HIV patients have been seen also. Historically, the diagnosis was made by staining and direct visualization of the organism within the bronchoalveolar lavage (BAL) fluid. The diagnosis is now made by microscopic examination and a real-time polymerase chain reaction (PCR) of BAL. Serum (1,3)-β-D-glucan, which is a component of the *Pneumocystis jirovecii* cell wall that distinguishes it from other fungi, has become an important diagnostic tool. Early diagnosis and treatment play a vital role in the patient’s survival and in the infection outcome; hence, empirical PCP therapy should be started immediately when the infection is suspected without waiting for the results of the diagnostic test. Steroids play an important role in the treatment of HIV patients, especially patients who present with hypoxia and respiratory failure. Prophylaxis is very effective and should be given to all patients at high risk of PCP. Antiretroviral therapy (ART) should be started as soon as possible in newly diagnosed HIV-infected patients with PCP, and the immune status of immunocompromised patients with PCP should be improved by temporarily withholding immunosuppressive drugs or reducing their doses.

## Introduction and background

*Pneumocystis* pneumonia (PCP) is caused by a ubiquitous unicellular fungus that is found all over the world. Originally thought to be a single protozoan species that infect a wide range of mammalian hosts, it is now a genus of fungi that is composed of a very diverse group of species with apparently strong host species specificity. The species that infects humans is *Pneumocystis jirovecii* [1]. Infection with *P. jirovecii* cysts is airborne and can be transmitted between humans. Its DNA can be detected in the air around PCP patients and asymptomatic carriers. This suggests that *Pneumocystis jirovecii* is being exhaled by them [2]. The first case of PCP was observed in the 1960s as a disease caused by the presence of opportunistic pathogens in children with congenital T-cell immunodeficiency and patients with hematological malignancies [3]. In 1981, five cases of PCP in previously healthy young gay men in Los Angeles were reported. This was the first time the acquired immunodeficiency syndrome (AIDS) had been identified [4]. Although PCP was rarely explained before the human immunodeficiency virus (HIV)/AIDS epidemic, the incidence of PCP increased rapidly in the 1980s, with PCP occurring in 75% of AIDS patients [5]. According to the most recent reports, more than 400,000 cases of human immunodeficiency virus patients develop PCP every year [6]. Mortality from all causes of PCP ranges from about 6%-11% but can be as high as 50% depending on the degree of immunosuppression [7]. The most common symptoms are dyspnea, unproductive cough, and subacute onset of low-grade fever. Patients generally suffer from tachycardia and undergo a normal lung examination. In particular, HIV-negative patients usually have more abrupt symptoms and more severe clinical symptoms than HIV-positive patients [1]. Trimethoprim-sulfamethoxazole (TMP-SMX) is the treatment of choice for adults and children. Alternative treatment regimens include dapsone with trimethoprim, clindamycin with primaquine, atovaquone, or pentamidine [5].

Conversely, the increasing incidence of PCP in non-HIV immunocompromised patients in recent decades is due to allogeneic bone marrow or organ transplantation, malignancies, corticosteroids, or other immunosuppressive drugs for inflammatory diseases [8]. Non-HIV immune-vulnerable patients with PCP infection are at significant risk of morbidity and mortality [8]. In the United States, the incidence of *Pneumocystis* pneumonia in HIV-infected individuals has dramatically decreased during the era of antiretroviral therapy (ART). The incidence of PCP in HIV patients has been less than one per 100 persons since the year 2000. However, PCP is still the second most commonly diagnosed opportunistic disease that defines AIDS. Even in developed countries with quality medical care, it is still a life-threatening disease [9].

Search strategy

The following keywords were used: *Pneumocystis* pneumonia, HIV, non-HIV, treatment, and prophylaxis. Articles that analyzed the connection between *Pneumocystis* pneumonia, HIV, and other immunocompromised conditions were searched using PubMed and PubMed Central (PMC) as the primary database. Various studies used to collect data were analyzed, including case reports, literature reviews, clinical trials, retrospective studies, systematic reviews, and meta-analyses. There were no demographic, age, or ethnicity limitations in the search. Articles published only in English were included, and all other articles translated into other languages ​​were excluded.

## Review

Structure and life forms of *Pneumocystis jirovecii*

PCP is caused by a ubiquitous unicellular fungus that is distributed worldwide. It was initially believed to be a single protozoan species due to its appearance under the microscope, as it was first identified in an animal that was infected with *Trypanosoma cruzi*. It is now unequivocally identified as a fungus. It is considered an atypical fungus based on numerous properties that differentiate it from other typical fungi, such as the absence of ergosterol in its cell wall. Comparably, the *Pneumocystis* fungus has cholesterol as its major sterol in the cell wall. Some genomic analysis shows that *Pneumocystis* fungus does not contain the same component characteristics of the cell wall of other fungi, including outer-chain mannans (high mannose residues of glycoproteins) and chitin, as mentioned. Therefore, *Pneumocystis* fungus is currently the only fungus that lacks chitin in its cell wall. *Pneumocystis* fungus has all the enzymes required for the biosynthesis and degradation of β1,3-glucan and β1,6-glucan, which are not present in other fungi. Another atypical feature of the *Pneumocystis* fungus is the inability of researchers to grow organisms in vitro, despite using other culture systems, including fungal media and coculture with mammalian cells [1].

There are many various species of the *Pneumocystis* fungus, and it can colonize a large number of healthy mammals. The organism is host-dependent and species-specific; it cannot grow outside the host, and the organism of one host cannot infect the host of other mammals [10]. Five different *Pneumocystis* fungus species have been discovered: *Pneumocystis carinii* and *Pneumocystis wakefieldiae *in rats, *Pneumocystis murina *in mice, *Pneumocystis oryctolagus *in rabbits, and *Pneumocystis jirovecii *in humans. *Pneumocystis* fungus occurs in two different forms during its life cycle, each with a distinct morphological appearance. A trophozoite forms a proliferative stage, an asexual stage of the life cycle, and a cyst form represents the productive stage. Cysts are produced during the sexual stage as a result of vegetative conjugation [3].

The source of infection is not known yet, but some scientists suggest that it is transmitted from host to host as an aerosolized particle. Therefore, the study of its life cycle has been a huge challenge over the last few years. The *Pneumocystis* fungus shows a high affinity for alveolar epithelial cells; once inhaled, the trophic form uses the extracellular matrix protein, principally fibronectin, and vitronectin to bind to type 1 alveolar cell. However, some rare cases of extrapulmonary dissemination have been found in patients with severe immunosuppression. Disorders in CD4+ T-cell-mediated immunity make the host more susceptible to *Pneumocystis* infection. Nonetheless, the innate immune system also contributes to host defense mechanisms by limiting the growth of fungi in the immunocompromised hosts [11].

Current epidemiology and risk factors

Despite the presence of effective chemoprophylaxis, the current global estimate is that there are up to 500,000 cases per year with a mortality rate of 10%-30%. Detailed knowledge of PCP epidemiology is very important for optimizing current preventive strategies. However, few studies have been conducted in recent years to provide data on the epidemiology of PCP [12]. PCP was rare in the United States; between November 1967 and December 1970, 194 cases were reported to the Centers for Disease Control and Prevention, with less than 100 cases per year in the United States in the 1970s. At the beginning of the AIDS epidemic in the United States, PCP was responsible for two-thirds of hospitalized patients with AIDS-defining disease [5,10].

PCP mainly occurs in people living with HIV patients in whom the number of CD4+ lymphocytes (CD4 count) in peripheral blood is less than 200 cells/μL or less than 14% of all lymphocytes [13]. In recent years, the use of ART drugs has reduced the incidence of PCP among HIV patients, but the incidence of PCP continues to increase due to advances in cancer treatment and other immunodeficiency disorders, which leads to significant changes in the patient’s survival and PCP incidence [14]. According to several studies in the United States, PCP gradually decreased from 6.7 in 2002 to 3.5 in 2014, thanks to significant improvements in primary care and widespread use of these drugs [4]. All those changes have led to other groups of patients who are susceptible to PCP infection, such as patients with hematological malignancies, especially patients with acute lymphoblastic leukemia, non-Hodgkin’s lymphoma, and chronic lymphocytic leukemia. Interestingly enough, some of the chemotherapeutic agents used to treat these malignancies also increase the risk of PCP infection; some of these drugs include methotrexate, vincristine, cytarabine, fludarabine, rituximab, and prolonged use of corticosteroids. According to some studies on this group of patients, this represents nearly one-third of PCP cases [15].

An additional subset of patients who are at high risk of PCP infection in patients with inflammatory and autoimmune disorders include patients with polymyositis, Wegener’s granulomatosis, dermatomyositis, polyarteritis nodosa, and rheumatoid arthritis. The risk in this group is much higher with the coadministration of immunosuppressant drugs such as methotrexate, antitumor necrosis factor (TNF) alpha, and anti-interleukin (IL)-6 receptor antibody, considering the risk is also higher for male patients and patients above 60 years old [16]. Children and adolescents are also at risk of developing PCP infection, especially those patients with gene mutations that affect T-cell function, most commonly in patients with severe combined immunodeficiency and adenosine aminase mutation disorders. PCP is additionally considered a challenging opportunistic infection to hematopoietic stem cell transplant patients and solid organ transplant recipients, as the treatment plan includes steroids and other immunosuppressive drugs; therefore, particular attention should be paid to this group of patients [15]. In older patients over the age of 60, PCP was more common in non-HIV patients compared to HIV patients. This is because homosexual activity and drug injections are less common in people over the age of 60. Older patients have been much more likely to have comorbid diseases (non-HIV conditions) such as cardiovascular and renal diseases. Under the age of 60, PCP is more common in HIV patients as increased homosexual activity increases the number of young men diagnosed with HIV [17].

Clinical representation and diagnosis

Symptoms and Signs

The traditional triad of PCP signs and symptoms in AIDS are the subacute onset of exertional dyspnea, dry and nonproductive cough, and fever. In HIV patients, the subacute path over numerous days or even weeks regularly permits differentiation from bacterial pneumonia. Oral thrush and full-size weight reduction also are regularly visible in HIV patients with PCP, as proven in our patients. Despite the subacute path, deterioration can also additionally arise rapidly. In HIV-negative patients, PCP progresses rapidly over fewer days. Respiratory failure and hypoxia are also more frequent [5]. The patient is hypoxic if the oxygen saturation is below 95%, and respiratory failure is rated positively if the patient needs positive pressure ventilation support, regardless of either invasive or noninvasive, to keep their ventilation and blood oxygen level normal [18]. A chest X-ray may show bilateral hilar infiltrates, especially in patients with markedly decreased CD4+ T-cell count. The initial chest image may be normal in HIV-negative patients due to the acute onset of the disease. A high-resolution CT scan of the chest is recommended. This allows the radiographic image to be accurately described to distinguish it from other lung diseases. The most common radiological pattern of PCP on CT is bilateral ground-glass opacity. Less common patterns include cysts and consolidation, mainly in HIV patients [5].

Diagnosis

Rapid diagnosis of PCP is important for serious symptoms that can be avoided with the early start of treatment, especially in non-HIV patients. This is due to the low fungal load, which makes the diagnosis difficult and leads to high false-negative results. It has been shown that shortening the start of treatment to just one day significantly reduces mortality [3]. Another feature that distinguishes *Pneumocystis* species from other fungi is the lack of a proper system for in vitro culture. Consequently, the diagnosis mainly depends on the visualization of PCP under the microscope. Previously, diagnostics relied on the visualization of the cyst and the trophic form of the respiratory sample. Direct or indirect immunofluorescence assay is the most sensitive microscopic method and advanced to standard staining methods. If possible, bronchoscopy with bronchoalveolar lavage (BAL) has to be performed because the evaluation of BAL fluid now no longer offers a simple, better diagnostic yield, yet it permits the exclusion of opportunity diagnoses or coinfections (e.g., tuberculosis (TB), histoplasmosis, and cytomegalovirus (CMV)). Induced sputum with hypertonic saline is a less invasive alternative but has a low sensitivity of 55%-90% [5]. However, there are some limitations to such visualizations, including samples taken during sputum induction that some patients may not tolerate or from the invasive method BAL and lung biopsy. Moreover, histological detection depends primarily on the observer’s experience and is time-consuming. Ultimately, low fungal loads that interfere with microscopic observation can lead to false-negative diagnoses, especially in non-HIV patients [3].

Several molecular strategies were examined as an approach to diagnose *Pneumocystis* from BAL or sputum samples. One such approach evolved in the 1990s into the usage of a single-round polymerase chain reaction (PCR) to increase the mitochondrial small subunit rRNA of *Pneumocystis*. Since then, nested PCR (two-round PCR) has been used for multiple gene targets. Using a nested approach increases sensitivity to almost 100%, but at the expense of reduced specificity, as it can detect colonized individuals who may not be active. Quantitative real-time PCR has now widely replaced previous methods, increasing specificity by being able to differentiate between truly infected cases from colonized individuals without infection [5]. One of the retrospective studies compared the accuracy of PCR and immunofluorescence antibodies (IFA) in diagnosing PCP for HIV using BAL sampling while taking time and cost into account. IFA was cheaper and less time-consuming compared to PCR; that being so, IFA can be used as a gold standard for the diagnosis, especially in HIV patients. However, in some cases with low PCP load, mainly in non-HIV patients, IFA showed low sensitivity. Therefore, the highly sensitive PCR can be used instead [19]. Table 1 compares the use of PCR with IFA in the diagnosis of PCP.

**Table 1:** Comparison of the use of PCR with IFA in the diagnosis of PCP in 66 samples collected from 62 patients The table was created by the first author Mohamed Nasr. The idea of the table was granted from the article “Clinical characteristics, treatment outcomes, and prognostic factors of Pneumocystis pneumonia in non-HIV-infected patients” [18]. PCR: polymerase chain reaction, IFA: immunofluorescence assay

Test	PCR	IFA
Positive test result	31	30
Negative test result	35	36
Sensitivity	100%	100%
Specificity	97.2%	95.8%

**Figure 1:**
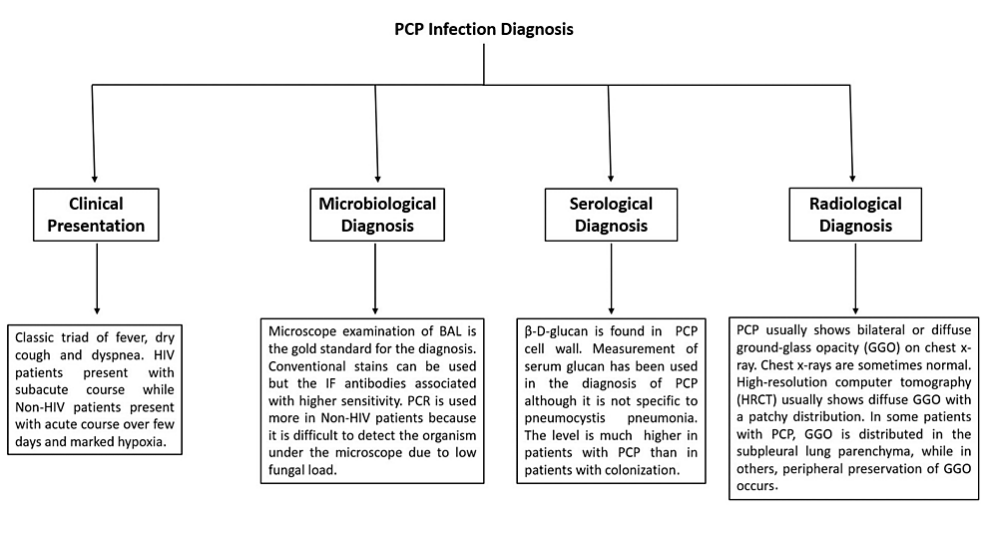
Summary of the diagnosis of PCP The figure was created by the first author Mohamed Nasr. The idea of the figure was granted from the article “Recent Advances in the Diagnosis and Management of Pneumocystis Pneumonia” [2].

The measurement of the cell wall component (1,3)-β-D-glucan in the serum has become an important diagnostic tool ascribed to its high negative predictive value and reduces the likelihood of PCP infection in patients with negative test results. However, it should not be used as a stand-alone diagnostic test for PCP, and a positive result should encourage bronchoscopy with BAL [5]. An increase in serum lactate dehydrogenase has been observed in patients with PCP, but this finding is not specific and is seen in other respiratory and non-respiratory diseases. At the onset of infection, hyperventilation results in normal or near-normal arterial oxygen partial pressure. As the infection progresses, hypoxia begins to appear, and the alveolar-arterial oxygen gradient increases [10]. Figure 1 summarizes how to diagnose PCP.

Treatments and prophylaxis

Empirical therapy can be started in HIV patients presenting with typical symptoms and radiological findings of PCP, especially if the patient is not under ART and CD4 T and the cell count is markedly decreased, given that the probability of other infections is low [10]. The first-line treatment for PCP is trimethoprim-sulfamethoxazole (TMP-SMX). The drug is available in both oral and parenteral forms. The endorsed length of treatment is 21 days in HIV patients and 14 days in non-HIV immunocompromised hosts. The recommendations for longer treatment in HIV patients are primarily based on high organism load and slowly progressive course, which may lead to a high chance of relapse if the drug is used only for 14 days. The recommended daily dose is 15-20 mg/kg trimethoprim and 75-100 mg/kg sulfamethoxazole [2]. Unfortunately, this drug cannot be tolerated by all patients due to its serious side effects such as bone marrow suppression, elevated liver enzymes, fever, rash, and gastrointestinal upset; dose reduction is associated with not only decreasing toxicity but also decreasing efficacy. Moreover, folinic acid, which is used to prevent bone marrow suppression associated with methotrexate, and its coadministration in PCP patients are associated with increasing the chance of treatment failure. Some alternative drugs used in PCP treatment include clindamycin, primaquine, atovaquone, and parenteral pentamidine, some of which are as effective as TMP-SMX and associated with low adverse reactions [10].

In case of hypoxia < 70 mmHg, steroids are added to the treatment regimen and are associated with reduced mortality in HIV-infected patients with PCP. Despite that, their use in non-HIV-infected patients with PCP is controversial, and some studies indicated that their use is associated with increased mortality [18]. One of the more serious problems associated with the treatment of PCP in HIV is immune reconstitution inflammatory syndrome (IRIS), in which the immune system starts to recover after the introduction of ART. The immune system then begins to respond to the infection with a severe inflammatory response that paradoxically exacerbates the symptoms of the infection and can display with a cough, dyspnea, and fever. Some studies have shown that low CD4+ T-cell counts and high viral load at the beginning of the treatment are the major risk factors for IRIS. Other studies suggest that the early use of ART is associated with the development of IRIS, but delayed adoption of ART is associated with an increase in *Pneumocystis*-related mortality [20].

TMP-SMX is the most commonly used agent for PCP prophylaxis. Furthermore, it offers safety in opposition to toxoplasmosis, nocardiosis, and actinomycosis. For those reasons, TMP-SMX is the first-desire agent for PCP prophylaxis. It is unknown whether or not the low doses of TMP-SMX used for PCP prophylaxis are also powerful in stopping different infections not applicable to variations in efficacy of various dosing techniques for TMP-SMX. If the drug has serious side effects or is intolerable to the patient, atovaquone, dapsone, or pentamidine can be used. Atovaquone is recommended as the first alternative to TMP-SMX for PCP prevention in patients with underlying hematological or oncological malignancies as this drug is very effective. This is because the drug is very potent and is not associated with bone marrow toxicity [21]. According to one study, patients with rheumatic disease treated with high doses of steroids above 60 mg/day may benefit from TMP-SMX prophylaxis, but the ideal time to discontinue prophylaxis remains unclear [22].

Caspofungin, one of the most common drugs in treating fungal infection, acting by inhibiting 1,3-β-D-glucan synthase, shows some efficiency in treating PCP in vitro, but its clinical use is controversial. Unlike TMP-SMX, which mainly acts against trophic forms, caspofungin eliminates the cystic forms, which can play an important role in transmission [23]. In HIV patients undergoing ART, the incidence of secondary PCP is 10/1,000 per year, as long as the CD4 cell count exceeds 95. The risk remains above 10/1,000 even with a high CD4 T-cell count, given that the virus is replicating because viremia is the leading cause of primary and secondary PCP. Secondary PCP prophylaxis can consequently be appropriately discontinued in asymptomatic patients on ART with plasma viral loads of <200 copies and CD4 T-cell count between 100 and 200 cells/µL. Primary prophylaxis can be safely discounted in patients with a CD4 count above 100 cells/μL and persistent viral suppression according to the current guideline in the United States and Europe [13].

Outcomes of PCP infection

Numerous studies have compared the outcome of PCP in HIV and non-HIV patients. One study showed that in less than 10 years, the proportion of non-HIV and HIV patients with PCP increased dramatically from 1.7 to 5.6. Most non-HIV patients received corticosteroids and chemotherapy, with the most common underlying disorders being hematological malignancies and cancer. At diagnosis, PCP was less symptomatic in non-HIV patients than in HIV-positive patients. The absence of symptoms is described as an independent predictor of mortality. In addition, it has been described that HIV-positive patients have a longer duration of symptoms compared to non-HIV patients. The study also showed that non-HIV patients have a higher mortality rate from PCP infection than HIV-positive patients. One of the reasons contributing to the high mortality in non-HIV patients is the high false-negative microscopic examination results due to low fungal load. Consequently, real-time PCR should be used to diagnose or rule out infections, even in patients who do not have any symptoms but have a risk factor [24].

There are some possible reasons for the poorer outcome of PCP in HIV patients, that being most of these patients are elderly patients and have many comorbidities such as end-stage renal and cardiovascular diseases. The time from the onset of symptoms to the start of PCP treatment is much longer in non-HIV patients. HIV-positive patients with PCP have benefited from adjunctive corticosteroid therapy; however, there is no evidence that adjunctive corticosteroid is useful to non-HIV patients. Non-HIV patients had a lower rate of emergency hospitalization than HIV-positive patients, and many HIV-positive patients showed signs and symptoms of acute respiratory failure. The study also showed that HIV-positive patients had extended inpatient stays than non-HIV patients [17]. One of the most common factors affecting the outcome of PCP infection is coinfection with CMV. Many studies have investigated the consequences of PCP and CMV coinfection and have shown that it is associated with adverse outcomes. One study compared the outcome of PCP infection and PCP+CMV infection in renal transplant patients. This showed that the mortality rate of the PCP+CMV group was twice that of the PCP group [25].

Limitations

The search strategy for this article review was limited to one, the PubMed database, searching for free full-text studies published in English in the last 10 years. Additionally, our search did not include numerous statistical analyses. This was a formal review of selected data from studies, including patients who had HIV and other immunosuppressive conditions, without focusing on any other risk factors.

## Conclusions

In recent years, great progress has been made in understanding HIV/AIDS and *Pneumocystis* pneumonia, but our knowledge of *Pneumocystis jirovecii* is still limited due to the restrictions of growing organisms outside the body. Since it is unclear why PCP infection is associated with a poor prognosis in non-HIV patients, further attention should be paid to this group, and additional research and studies should be done to identify the risk factors in non-HIV patients. The diagnosis of PCP in non-HIV patients is difficult because the course of the disease is different and the sensitivity of the gold standard test for diagnosis of PCP is low in non-HIV patients. Therefore, there is an urgent need for new diagnostic criteria for non-HIV patients and new guidelines for using the other highly sensitive tests in the diagnosis that contribute to the early detection of infection that can lead to improved mortality and better outcomes. PCP prevention is critical, but it is unclear when to start prevention, and further research is needed to determine the risk factors associated with increasing the risk of infection in HIV patients in order to determine the most appropriate time to initiate prevention.
